# Influence of Cotton Cationization on Pigment Layer Characteristics in Digital Printing

**DOI:** 10.3390/molecules27041418

**Published:** 2022-02-19

**Authors:** Martinia Ira Glogar, Tihana Dekanić, Anita Tarbuk, Ivana Čorak, Petra Labazan

**Affiliations:** Department of Textile Chemistry and Ecology, Faculty of Textile Technology, University of Zagreb, HR-10000 Zagreb, Croatia; martinia.glogar@ttf.unizg.hr (M.I.G.); anita.tarbuk@ttf.unizg.hr (A.T.); ivana.corak@ttf.unizg.hr (I.Č.); 5ra.labazan@gmail.com (P.L.)

**Keywords:** inkjet printing, cationization, cotton, pigment, wash fastness

## Abstract

The paper examines the influence of cotton cationization on the print quality in terms of penetration, colour yield and colour depth, which have been analysed in comparison to cotton untreated and pretreated with conventional acrylate binder. The process of cationization during mercerization was performed with a cationizing agent Rewin DWR (CHT Bezema). Standard (non-cationized) and cationized fabric, with and without additional layering of binder have been printed by digital inkjet pigment printing method. Moisture management testing (MMT) and dynamic contact angle measurement (drop shape analyzer–DSA30S) were performed on standard and cationized fabric, with and without binder, both with and without pigment layer. After printing, the objective values of colour depth (K/S) and colour parameters L*, C* and h° were analysed. The samples were also analysed by the method of microscopic imaging using a DinoLite microscope. Printed samples were tested to washing fastness, and the results are presented in terms of total colour difference (dECMC), according to CMC(l:c) equation, after the 1st, 3rd, 5th, 7th and 10th washing cycles. Results showed that the cotton cationization will improve the uniformity and coverage of the printed area as well as increase the K/S value. For the samples with binder, the positive effect of cationization on the stability and bond strength between the polymer layer as a pigment carrier with the cotton fabric was confirmed.

## 1. Introduction

Despite its unquestionable positive aspects, digital printing on textiles has not yet experienced its full commercialization and significant industrial application. According to a survey conducted by Zimmer in 2017, 97% of total world production of printed textiles is still carried out by conventional rotary printing technology [1]. The reasons for this are multiple and highly complex, arising from several contexts related to digital printing key issues: substrate characteristics, printing ink formulation, choice of dye or pigment, mechanism of interaction of printing ink droplets and textile substrate, technical requirements of machines and especially print heads for digital printing and requirements on the rheological properties of printing inks for digital printing. Currently, the most important directions of research in the field of digital textile printing is in innovative approaches in the printing ink formulation and modifications and pre-treatments of textile materials. In the application of pigments in printing ink formulation for digital textile printing, requests pertaining to pigment particle size, restricted content of binders and crosslinking agents in printing inks as well as their requested very low viscosity with higher surface tension opens an extensive platform for researching innovative formulations of binders, as well as innovative methods of textile surface pre-treatment [2,3,4]. Xue et al. experimented with the polymeric binders based on nanoscale emulsions containing different ratios of soft and hard monomers, coming out with the optimal results by applying a mixture of butyl acrylate soft monomers and methyl methacrylate hard monomers in ratio 8:1, employing, N-methylolacrylamide as crosslinking agents and monododecyl maleate as copolymerizable surfactant [5]. In the context of nanoscale binders, also, extensive research is being conducted in the field of chitosan application, as a pre-treating biomaterial [6,7]. As for the structural and chemical modification of a textile surface, in order to achieve optimal pigment layering and colour yield, with satisfactory fastness properties, plasma pre-treatment and cationic compounds have been used [8]. A review of the literature confirms the extensive research work in the field of application of cotton pre-treatment by cationization in digital printing processes based on dyestuff printing ink formulations [9,10,11]. Yang et al. [12] and Rekaby et al. [13] reported improved colour strength, increased colour yield, better pattern sharpness, higher colourfastness and reduced the steaming duration as well as washing-off procedure in reactive inkjet printing on cotton pre-treated by cationization. As for the cationic pre-treatment in pigment-based inkjet printing, the number of published studies is rather lower. Hauser and Kanik et al. studied printing of cationized cotton using reactive, direct and acid dye as well as pigments [14,15,16,17,18]. Wang and Zhang applied synergy of both cotton modification by commercially available cationic compounds Cibafix Eco and pigment modification, particularly the size of a pigment, achieving better colour strength and lower pigment penetration [19,20,21]. El-Shishtawy studied anionic dye and pigment printing. Cationization was applied as pre-treatment or after-treatment by exhaustion or padding and contributed to the better colour yield and fastness in all cases [22]. Grancarić, Tarbuk and Dekanić introduced cationization during mercerization, which results in cellulose with properties of both processes [23,24]. Application of the commercial long-chain cationic compounds in pre-treatment or after-treatment by exhaustion or padding, results in blocking of the surface active groups of cellulose, and if the dyeing process is performed afterwards, the coloration usually is not uniform. On the other hand, if the cationization is performed during the mercerization process, new cellulose is formed. Change in cellulose crystal lattice occurs and at the same time the cationic compound us evenly distributed and trapped between the cellulose chains, resulting in levelness of colour. There are no published papers related to printing on such modified cotton cellulose, especially ink-jet pigment printing and few papers related to dyeing with direct, acid, natural, vat and reactive dyes within same group of authors [25,26,27,28,29,30]. Therefore, the aim of this research was to investigate and analyse the pigment prints on samples of standard cotton fabric, untreated and modified by the cationization during the mercerization process considering binding of pigment ink and binder itself.

The aim of this work was to study the behaviour of the pigment-based inkjet print in dependence on the changes of substrate characteristics. The analyses were performed from the aspect of textile substrate pre-treatment, without interfering with constant and industrially defined settings of the printing process. Samples were digitally printed and the coverage of the textile surface with pigment, the uniformity of the pigment layer, the colour depth and objective colour parameters were examined. The wash fastness test was also performed and, in addition to objective evaluation, microscopic imaging of printed surfaces before and after washing was performed in order to determine the level of damage to the pigment polymer layer in the washing process, depending on fabric pre-treatment.

## 2. Materials and Methods

Standard fabric (WFK Switzerland, type 10A for ISO 2267) of 100% cotton was chosen, in canvas embroidery P1/1 with the following physical and mechanical characteristics:

- Standard, chemically bleached fabric, a flat weight of 170 g/m^2^, the density of the warp and weft was 27/27 threads per cm, and the fineness of the yarn was 295 dtex/295 dtex;
- Cationized cotton fabric during mercerization with Rewin DWR: mass per unit area of 185 g/m^2^ was measured, and the density of the warp and weft is 29/28.5 threads per cm.

The process of cationization during mercerization was performed according to [24] with a cationizing agent, Rewin DWR (CHT-Bezema, Montlingen, Switzerland). It was carried out at a continuous rate of v = 3 m/min, with 0% tenacity, at 20 °C, with 5 passes through the bath on a Jigger machine. First, the mercerization of 5 passages in 24% NaOH and 8 g/L of Subitol MLF (CHT-Bezema, Montlingen, Switzerland) was performed, followed by 5 passages in 50 g/L of Rewin DWR. Afterward, cotton fabric crosslinking occurs in a closed system for 24 h, followed by hot rinsing (fixation) with distilled water at 100 °C for 2 min. The cotton fabric is further rinsed, neutralized with 5% acetic acid (CH_3_COOH), followed by cold rinsing to achieve a neutral pH, and air-dried.

### 2.1. Fabric Surface Characterization

For the characterization of the changes of interface phenomena of the cotton fabric, zeta potential, absorption rate and contact angle were determined.

Electrokinetic potential (zeta, ζ) was measured using an electrokinetic analyser, EKA (Anton Paar, Graz, Austria). Measurement was performed according to the following conditions: sample mass = 0.2000 g; V_KCl_ = 500 mL; c_KCl_ = 10 − 3 mol/L; d = 0.55 mm (electrode spacing); p = 300 mbar; pH 2.5 to 10. The results of the measured zeta potential were calculated according to the Helmholtz-Smoluchowsky equation [30].

The moisture management test was performed according to AATCC 195-2020 *Liquid Moisture Management Properties of Textile Fabrics* on a moisture management tester (MMT). For testing, a solution prepared according to the producer specification was used: 9 g/L of NaCl on 1 L of distilled water, with the conductivity of 16 mS/ ± 0.2 mS. From measured properties, mean values of absorption rate (AR) and total ability to manage (liquid) moisture (OMMC) are presented, and type of fabric is given.

Measurements of the dynamic contact angle on the textile material surface were performed on a goniometer (Drop Shape Analyzer- DSA30S, Kruess, Germany) with the distilled water as test liquid.

### 2.2. Inkjet Printing

The printing of cotton samples was performed by digital inkjet technique on an Azon Tex Pro textile inkjet printer (Azon d.o.o. Zagreb, Croatia), with a micro piezo print head and aqueous pigment-based printing inks. The maximum print resolution of the machine is 1440 dpi. Machine properties and specifications are shown in Table 1. The printing inks were supplied under the commercial name Azon Pigment Ink, by Azonoprinter d.o.o. The composition of the printing ink is given in Table 2.

Both types of cotton fabrics (standard bleached and cationized) were printed without and with the addition of a surface acrylate-based binder–a polymer micro-emulsion ‘Pigment Pre-treatment Solution’ specially developed for digital printing by Azonprinter d.o.o. (Zagreb, Croatia). The composition of the binder used is shown in Table 3.

Samples were prepared in sizes of 20 cm × 30 cm, and consumption of the polymer micro-emulsion (binder) over defined surfaces were 30 mL.

Samples were printed in two primary colours, magenta and cyan. By software adjustment of the prepress, a print with two different amounts of pigment was defined: in full concentration to achieve maximum saturation, which would correspond to the software definition of 100% and in concentration to achieve a shade of lower saturation, and increased lightness, which would correspond to the software definition of 50% of a pigment.

### 2.3. Microscopic Imaging

The microscopic imaging of printed samples was performed using a DinoLite AM7013 microscope. The imaging was performed before and after the 10th cycle of washing process.

### 2.4. Colourfastness

Printed samples were tested to washing fastness, in a laboratory apparatus for wet and dyeing processes Polycolor, Mathis. The test was performed according to standard ISO 105-C06:2010 (A2S) Textiles—Tests for colour fastness—Part C06: Colour fastness to domestic and commercial laundering, using 1 g/L of Na_2_BO_3_H_2_O and 4 g/L of standard detergent (James Heal ECE A, free from optical brighteners and phosphates), with a bath ratio of 1:200, temperature of 30 ± 2 °C, time of 40 min and pH of 6.

The results of colour fastness have been obtained objectively by spectrophotometric measurement using a DataColor Spectra Flash 600 PLUS–CT remission spectrophotometer (with constant instrument aperture, D65, using d/8° geometry) and were graded according to grey scale. The results are presented in terms of total colour difference (dE_CMC_), according to the CMC(l:c) equation, accepted by ISO 105-J03:2009, for colour fastness and colour changes evaluation in the field of textiles [31]. The results were obtained after 1st, 3rd, 5th, 7th and 10th cycles of washing.

## 3. Results and Discussion

The purpose of cationic pre-treatment of cotton is to improve printability with pigment by introducing positively charged sites on cotton. Due to the pigment in the ink drop being ionized, the ink drops are negatively charged. Therefore, the ink drops could be immobilized on the cationized fabric surface. The print quality in terms of penetration, colour yield and colour depth have been analysed in comparison to untreated cotton and cotton pre-treated with conventional acrylate binder.

The aim of such research is to find an alternative to conventional binder application in order to achieve optimal colour properties, colour yield and colour fastness but with more satisfactory tactile and physical-mechanical fabric properties.

**Figure 1:**
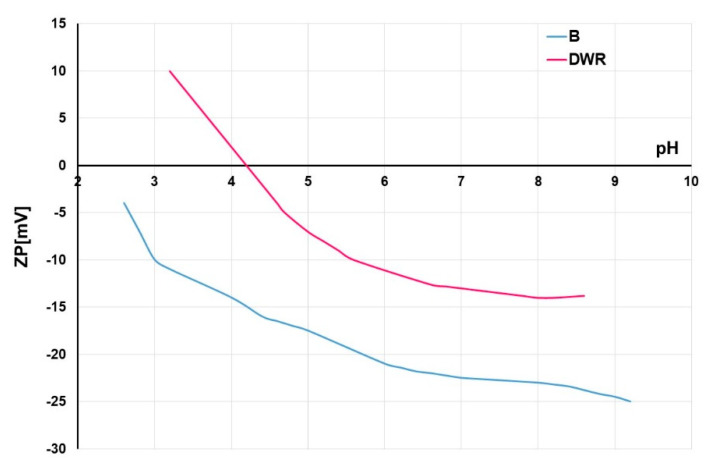
Zeta potential (ZP) vs. 0.001 mol/L KCl of cotton fabrics: standard unmodified (B) and cationized with Rewin DWR (DWR).

In order to compare standard and cationized cotton fabric from the aspect of surface charge and later analysis of the influence on the formation of surface pigment layer, the measurement of electro kinetic (zeta, ζ, ZP) potential was performed. In Figure 1, the zeta potential vs. pH of electrolyte 0.001 mol/L KCl is given.

Standard (unmodified) cotton fabric (B) has a negative zeta potential due to negative surface groups, hydroxyl of cellulose (-OH) and carboxyl that revels in chemical bleaching (-COOH). Therefore, ZP in whole pH range is negative, −24.7 mV at pH 9, and −10.0 mV at pH 3. No isoelectric point is present. Cationization during mercerization with Rewin DWR changes zeta potential. Ammonium groups (-NH_2_) from polyammonium compound are present as well, resulting in higher zeta potential of −14.5 mV at pH 9. A change of zeta potential towards more positive values is visible, and such cationized cotton has an isoelectric point at 4.2. Cationized cotton has a positive charge at pH lower than 4.2.

**Figure 2:**
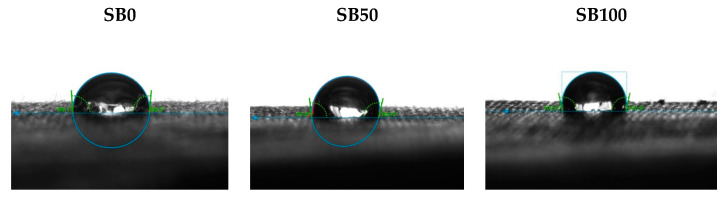
DSA images for samples without binder.

**Figure 3:**
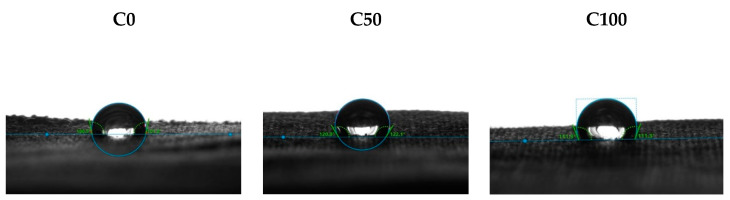

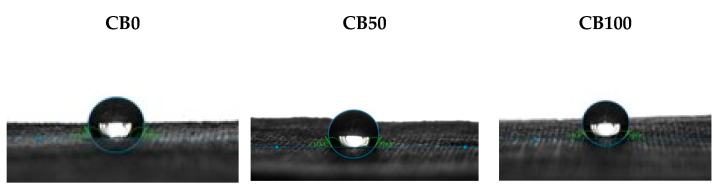
DSA images for samples cationized and cationized with binder.

The mechanisms of penetration and spread of droplets are fundamental mechanisms of ink and textile surface relationships in digital printing processes. Therefore, the MMT and contact angle measurement on DSA30S were performed. The results are presented in Table 4, Table 5, Table 6 and Table 7 and Figure 2 and Figure 3.

For this part of the analysis, the samples are labelled as follows:

1. Non-printed, non-treated standard fabric-S0; standard fabric printed with 50% pigment-S50; standard fabric printed with 100% pigment-S100; standard fabric pre-treated with binder, non-printed-SB0; standard fabric pre-treated with binder, printed with 50% pigment-SB 50; standard fabric with binder, printed with 100% pigment-SB 100;
2. Non-printed, cationized fabric without binder-C0; cationized fabric without binder, printed with 50% pigment-C50; cationized fabric without binder, printed with 100% pigment-C100; cationized fabric with binder, non-printed-CB0; cationized fabric with binder, printed with 50% pigment-CB 50; cationized fabric with binder, printed with 100% pigment-CB 100.

Since the printing was performed with a commercial, closed settings system of inkjet printing inks, the moisture management testing as well as the contact angle measurement were performed with the aim of characterization of changes in substrate to be able to better explain the influence of textile pre-treatment on the formation, capillary spread and penetration of droplets into the structure of the material, as well as the passage of droplets through the material.

Moisture management testing is carried out to determine the absorbency, water repellence and water resistance of the fabric, which largely depends on the fabric structure itself, but also on the pre-treatment of the fabric. A comparative measurement of standard and cationized samples was performed, with and without the addition of a binder and with and without the presence of a pigment layer. In Table 4, the absorption rate (AR) and the total moisture management capability (OMMC) values are presented as indicators of moisture management. Considering evaluation as type of fabric, non-binder-treated samples (S0, S50, S100) are classified as fast absorbing and quick drying fabric, regardless of whether they are printed (regardless of the pigment presence in the surface structure of the fabric). In the printing process of such surfaces, due to the pronounced capillarity of the untreated textile, rapid absorption of the aqueous phase occurs. There is a deeper penetration of pigment into the substrate, and it does not remain on the surface but is unevenly incorporated into shallower and deeper layers of yarn structure and is not a factor in moisture management. Increased absorption capacity is observed in the S100 sample (sample printed with the maximum amount of pigment).

For samples pre-treated with binder, a change in the absorption rate is observed. The standard non-printed sample with binder SB0 and sample printed with 50% of pigment content SB50 belong to the group of textile substrates with the ability to penetrate moisture, while the sample SB100 belongs to the group of water-repellent substrates.

Drop shape analyser (DSA) measures the contact angle of the droplet on the surface of the fabric and the time of absorption/spillage of the droplet from the surface. The results of DSA show the significant influence of binder pre-treatment. As can be seen from Table 5, droplet analysis for standard samples without binder could not be performed, due to too fast passage of the test droplet through the fabric. This result correlates to MMT overall evaluation of fast absorbing and quick drying fabric. Only pre-treatment with a binder gives a slower absorbing surface that retains the droplet for a certain time. The contact angle of the droplet with the fabric surface is shown for samples pre-treated with binder (Figure 2).

In contrast to non-cationized samples, Table 6 immediately shows that all three cationized samples without the binder (C0, C50 and C100) belong to the group of textile substrates with the ability to manage moisture. It corresponds to the effect of simultaneous mercerization and cationization, whereby a larger number of active groups are formed in cotton and the sample can bind a larger amount of water. A significant value of the absorption rate was obtained for T (upper surface), which confirms the importance of cationization. The application of pigment reduces the number of polar molecules in cotton, responsible for binding water, and it is seen by the results obtained for the printed cationized samples (C50 and C100). Cationized samples pre-treated with binder (CB0, CB50, CB100), according to the results obtained (Table 6), are classified as water penetration to water repellent fabric, suggesting strong binding of acrylic binder. Results of DSA correspond to and confirm the MMT results. The obtained high contact angle indicates that the surface has low wetting properties.

**Figure 4:**
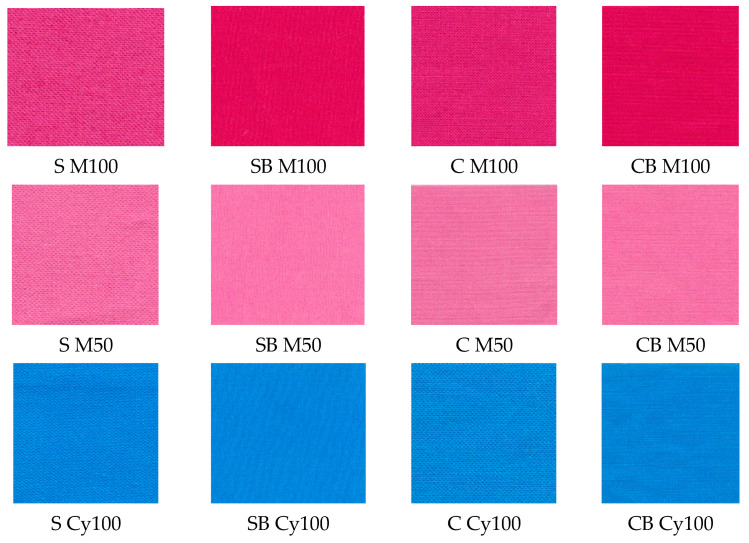

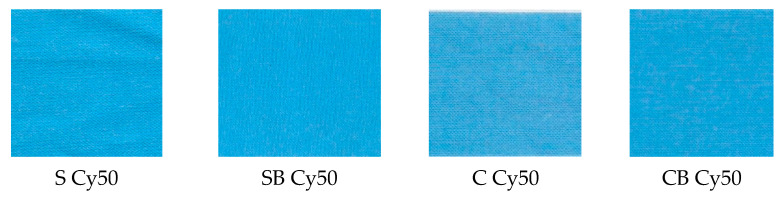
The appearance of printed samples.

In the next step, the samples were printed by digital inkjet technique of textile printing in two primary colours of the CMYK system, magenta and cyan, and the pigments were layered in concentrations of 50% and 100%. The appearance of samples is shown in Figure 4. Images of samples shown before washing confirm the positive effect of cationization of the cotton surface, on the coverage of the surface with pigment and the amount of bound pigment and the uniformity of the pigment layer. Even in the samples on which the conventional acrylate-based binder was applied, it is observed that the pre-treatment by cationization contributed to the achievement of greater coverage and more even distribution of the pigment layer. For the analysis of printed samples, unlike the previous analysis which included non-printed samples, new code abbreviations—labels are listed in Table 8 considering the pigment and pre-treatment.

**Figure 5:**
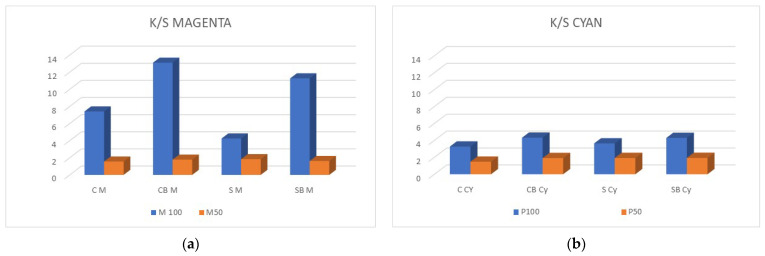
The K/S values of printed samples before washing. (**a**) Magenta and (**b**) Cyan pigment.

In Figure 5, the value of the colour depth coefficient K/S for samples before washing is shown. The highest colour depth (K/S value) was obtained for samples treated with a binder, with the positive effect of cationization being obvious. However, the relevant indicators of the cationization effect can be seen by comparing the samples that were not treated with the binder, and for the cationized samples, a higher K/S is obtained, which indicates a higher amount of bounded pigment.

Table 9 and Table 10 show the objective values of lightness (L*), chroma (C*) and hue (h*) of the measured samples. For the purpose of comparing the values of the colour parameters, the tables comparatively show the results for the non-cationized (labelled S) and cationized (labelled C) samples. In general, it can be seen from the results that the specific ratio of lightness and chroma, from which the colour intensity arises, depends on the interaction of the amount of pigment applied and the characteristics (pre-treatment) of the fabric.

The dependence of the results on the specific nature of the colour itself is also visible. For samples printed in magenta pigment (Table 5), the lightness (L*) of a standard sample without a binder printed with 100% pigment is lower, and the chroma (C*) is higher compared to the cationized fabric. This can be explained by a more uniform coverage of the cationized surface with pigment. There is less scattering of incident light and during the measurement the objective value of lightness (L*) is higher and the chroma (C*) is lower. Also, one should take into account the nature of the magenta colour itself, which is a colour of medium lightness and takes on maximum chroma at medium levels of lightness. For samples with added binder, the lightness (L*) and chroma (C*) results for standard and cationized fabric, for both pigment concentrations, were uniform.

In the case of samples printed with pigment in cyan hue, a more pronounced influence of cationization is observed, and higher values of chroma (C*) are obtained in all cationized fabric, regardless of the pre-treatment with binder.

**Figure 6:**
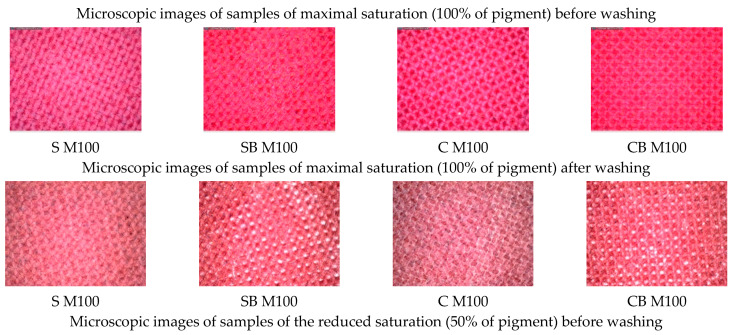

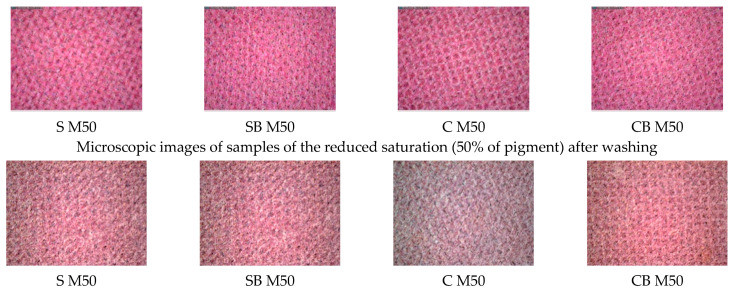
Microscopic images of samples printed in magenta colour hue, before and after the 10th washing cycle.

**Figure 7:**
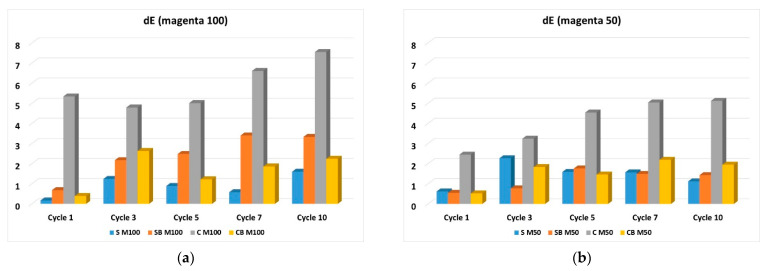
Total colour differences (dE) for washed samples compared with samples before washing, for magenta colour hue: (**a**) 100% (**b**) 50% of pigment.

**Figure 8:**
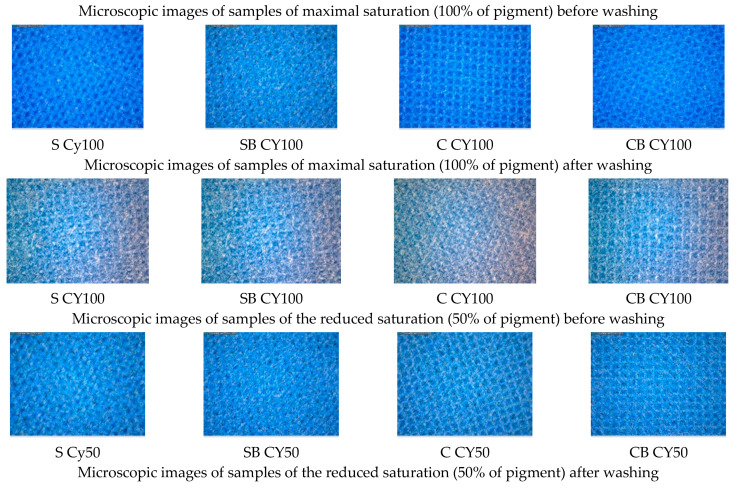

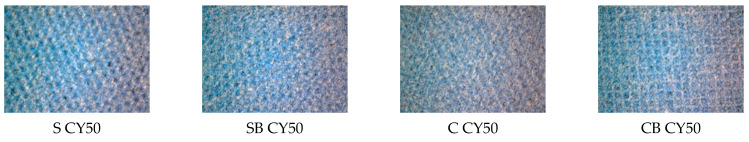
Microscopic images of samples printed in cyan colour hue, before and after the 10th washing cycle.

**Figure 9:**
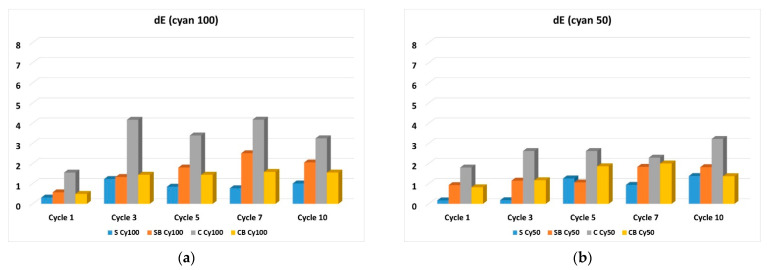
Total colour differences (dE) for washed samples compared with samples before washing, for cyan colour hue: (**a**) 100% (**b**) 50% of pigment.

In the further work, the test of resistance to washing was performed, which was carried out in standard washing conditions in 10 cycles. Due to the extensiveness of the analysis and the amount of results, the microscopic images of the samples before and after the 10^ć^ wash cycle will be presented, while the analysis in colour change in terms of total colour difference are shown for all washing cycles performed. The microscopic images for samples printed in magenta hue are shown in Figure 6, while the total colour differences (dE_CMC_) are shown graphically in Figure 7. Also, the images of samples printed in cyan hue are shown on Figure 8 and the total colour differences for cyan-printed samples are shown in Figure 9.

Comparative analysis of microscopic images of samples printed with 50% and 100% pigment content confirmed that the cationized cotton achieves a more uniform pigment layer, higher colour efficiency with less pigment penetration into the textile structure, which is the goal to keep the pigment on the surface while preserving natural physical and mechanical properties of textile fabric.

Also, visual analysis of the washing effect reveals, in general, lower damage of the pigment layer in cationized fabrics. Namely, in the case of binder-free samples, there is a deeper penetration of pigment within the fabric structure. The influence of the surface structure of textiles is more pronounced, resulting in darker and less saturated colours. By applying the binder, a cross-linked three-dimensional polymer structure is created on the surface of the fabric, which keeps the pigment on the surface, and the entire layer is thicker, more even and the influence of the structure is less pronounced. However, due to the retention of the pigment layer on the textile surface, in samples with the addition of binders, both for standard and cationized fabric, there is more damage in washing cycles because there is cracking and peeling of the polymer layer. It is in these samples, with binder, that less damage and greater stability of the pigment layer is observed in cationized fabrics. This is because conventional binders of the older generation are of anionic character, and cationization of the fabric promotes the creation of a stronger bond between the polymer layer of binder and pigment with the fabric.

A comparison of the total differences (dE) as an analysis of the changes that occur in the pigment layer during the wash cycles shows the relative unevenness of the results. By comparing samples without binder, higher colour differences are obtained for cationized fabrics. The cationization of cotton led to the deposition of more pigment, and as expected, there was a significant release in the washing process, especially since there is no additive binder. However, in the case of samples with binder, printed with a higher amount of pigment (100%), lower values of the difference are obtained for cationized fabrics compared to non-cationized ones. For samples printed with a lower pigment concentration (50%), the results are uneven, and a detailed comparison cannot be performed. This is partly due to the fact that magenta pigment is a mixture of red and blue pigment. With lower amounts of pigment, i.e., with lighter colours, uneven removal of pigment occurs during washing, and there are significant changes in colour, which can be seen in microscopic images. Therefore, high values of the total colour difference will be the result of significant shifts in colour tone, and such differences cannot be attributed solely to lower fastness, but to certain changes in the spectral characteristics of colour.

In samples printed with pigment in cyan hue (Figure 8), the same effect of more uniform pigment layer and more even surface coverage in cationized fabrics is observed, in general, regardless of the amount of pigment and pre-treatment with binder. Also, less damage of the pigment layer due to washing is observed. Although samples printed with 100% pigment (more saturated colour of the print) show a significant change in colour after the 10th wash cycle, and obviously a certain amount of pigment is removed, the damage shown on microscopic images in the form of peeled parts of the polymer layer is less in cationized samples.

Also, in cyan, the lower wash fastness of the pigment layer is confirmed in cationized fabrics without binder compared to the non-cationized ones, but in samples with added binder, generally better pigment layer stability and wash fastness was obtained for cationized samples (Figure 9).

## 4. Conclusions

In the initial printing phase, the K/S values confirmed the obtained higher values of colour strength of cationized fabrics. The reason for the colour strength increase is that, as the cationic reagent concentration increases, the ionic attraction of the anionic pigment by the fibre’s cationic charges also increases. The cationization results in better penetrating properties because of the reaction between the hydroxyl group of cotton and the cationic reagent. The wash colourfastness phase did not fully confirm the positive effect of cationization on the bonding of the pigment to the cotton fabric, but in the samples with binder, the positive effect of cationization on the stability and bond strength between the polymer layers as a pigment carrier with the cotton fabric was confirmed. This research is part of the extensive work that continues in the field of testing innovative methods of cotton processing in digital pigment printing processes.

## Figures and Tables

**Table 1:** Inkjet textile printing machine technical characteristics.

Print Head	Nozzle (Colour)	Nozzle (White)	Ink Cartridge	Max. Ink Drop Size	Print Speed
Piezo	192/per Colour	192 × 4	200 mL (with encrypted chip)	Up to 37 pL	Fast-28 s (C)/55 s (C + W) Normal-53 s (C)/102 s (C + W) Quality-79 s (C)/165 s (C + W)

**Table 2:** Printing inks composition.

Colour	Water (%)	Aliphatic Alcohol (%)	Ethylene Glycol (%)	Polyglycol Ether (%)	Polymers (%)	Pigment (%)	Triethylene Glycol Monobutyl Ether
Yellow	55–95	1–10	1–10	1–10	1–10	1–5	
Magenta	50–94	1–10	1–10	1–10	1–10	1–5	1–5
Cyan	55–95	1–10	1–10	1–10	1–10	1–5	
Black	55–95	1–10	1–10	1–10	1–10	1–5	

**Table 3:** Composition of binder used.

Components:	Water	Inorganic Nitrate	Acrylic Polymer	Formaldehyde
Ratio (%):	65–85	10–20	5–15	<0.02

**Table 4:** The results of moisture management testing for standard (non-cationized) cotton fabric presented as mean value.

		S0	S50	S100	SB0	SB50	SB100
AR (%/s)	T	44.03	45.00	71.67	89.13	41.07	112.09
	B	55.30	71.00	69.33	47.40	54.32	20.25
OMMC		0.50	0.43	0.37	0.32	0.37	0.04

Symbols: T-upper surface; B-lower surface; AR-absorption rate; OMMC-total ability to manage (liquid) moisture.

**Table 5:** Drop shape analyser (DSA) results for standard (non-cationized) cotton fabric.

S0		S50		S100		SB0		SB50		SB100	
CA	T	CA	T	CA	T	CA	T	CA	T	CA	T
/	/	/	/	/	/	49.76	42.00	76.82	28.00	81.16	15.00

Symbols: CA (m)-contact angle mean value; T-droplet absorption/spill time in seconds (s).

**Table 6:** The results of moisture management testing for cationized cotton fabric presented as mean value.

		C0	C50	C100	CB0	CB50	CB100
AR (%/s)	T	119.76	23.59	35.04	107.71	58.83	118.61
	B	17.51	29.10	40.18	18.35	48.88	29.24
OMMC		0.24	0.37	0.32	0.02	0.29	0.18

Symbols: T-upper surface; B-lower surface; AR-absorption rate; OMMC-total ability to manage (liquid) moisture.

**Table 7:** DSA results for cationized cotton fabric.

C0		C50		C100		CB0		CB50		CB100	
CA	T	CA	T	CA	T	CA	T	CA	T	CA	T
75.99	43	95.01	16	86.84	9	79.32	68	86.90	43	92.74	31

Symbols: CA (m)-contact angle mean value; T-droplet absorption/spill time in seconds (s).

**Table 8:** Sample descriptions and labels with code abbreviation.

Sample Descriptions	Label	
	Magenta	Cyan
Standard fabric, no binder, full pigment concentration (100%)	S M100	S Cy100
Standard fabric, no binder, full pigment concentration (50%)	S M50	S Cy50
Standard fabric, with binder, full pigment concentration (100%)	SB M100	SB Cy100
Standard fabric, with binder, full pigment concentration (50%)	SB M50	SB Cy50
Cationized fabric, no binder, full pigment concentration (100%)	C M100	C Cy100
Cationized fabric, no binder, full pigment concentration (50%)	C M50	C Cy50
Cationized fabric, with binder, full pigment concentration (100%)	CB M100	CB Cy100
Cationized fabric, with binder, full pigment concentration (50%)	CB M50	CB Cy50

**Table 9:** Objective values of colour parameters lightness (L*), chroma (C*) and hue (h°) for samples printed with magenta pigment.

		L*		C*		h°	
S M100	C M100	45.29	50.51	50.08	44.55	352.75	349.6
S M50	C M50	61.53	60.24	31.53	34.51	349.11	348.92
SB M100	CB M100	40.25	41.36	54.78	54.21	358.78	359.12
SB M50	CB M50	60.68	61.79	34.20	33.52	349.07	349.11

**Table 10:** Objective values of colour parameters lightness (L*), chroma (C*) and hue (h°) for samples printed with cyan pigment.

		L*		C*		h°	
SCy100	C Cy100	55.54	54.89	35.9	37.74	248.71	248.22
S Cy50	C Cy50	64.66	63.26	26.62	30.97	234.48	233.29
SB Cy100	CB Cy100	53.07	53.89	37.63	39.06	245.77	245.93
SB Cy50	CB Cy50	62.06	62.95	29.11	31.05	234.30	234.57

## Data Availability

Data available in a publicly accessible repository.

## Sample Availability

Samples of the compounds are available from the authors.
